# Paradigm shift: contribution of field epidemiology training in advancing the “One Health” approach to strengthen disease surveillance and outbreak investigations in Africa

**Published:** 2011-12-15

**Authors:** Busuulwa Monday, Sheba Nakacubo Gitta, Peter Wasswa, Olivia Namusisi, Aloysius Bingi, Monica Musenero, David Mukanga

**Affiliations:** 1 African Field Epidemiology Network (AFENET), Kampala, Uganda

**Keywords:** Field epidemiology, training, one health, disease surveillance

## Abstract

The occurrence of major zoonotic disease outbreaks in Sub-Saharan Africa has had a significant impact on the already constrained public health systems. This has, as a result, justified the need to identify creative strategies to address threats from emerging and re-emerging infectious diseases at the human-animal-environmental interface, and implement robust multi-disease public health surveillance systems that will enhance early detection and response. Additionally, enhanced reporting and timely investigation of all suspected notifiable infectious disease threats within the health system is vital. Field epidemiology and laboratory training programs (FELTPs) have made significant contributions to public health systems for more than 10 years by producing highly skilled field epidemiologists. These epidemiologists have not only improved disease surveillance and response to outbreaks, but also improved management of health systems. Furthermore, the FETPs/FELTPs have laid an excellent foundation that brings clinicians, veterinarians, and environmental health professionals drawn from different governmental sectors, to work with a common purpose of disease control and prevention. The emergence of the One Health approach in the last decade has coincided with the present, paradigm, shift that calls for multi-sectoral and cross-sectoral collaboration towards disease surveillance, detection, reporting and timely response. The positive impact from the integration of FETP/FELTP and the One Health approach by selected programs in Africa has demonstrated the importance of multi-sectoral collaboration in addressing threats from infectious and non- infectious causes to man, animals and the environment.

## Introduction

In the last decade, sub Saharan Africa has recorded major zoonotic disease outbreaks consisting of deadly diseases such as Ebola, Marburg, yellow fever, rift valley fever, plague, anthrax, Lassa fever, pandemic influenza A H1N1, rabies, and brucellosis. Non- zoonotic diseases such as acute diarrheal diseases (typhoid fever, cholera) are also common [1].

Evidence shows that of the almost 1500 diseases now recognized to affect humans, approximately 60% are due to multi-host pathogens characterized by their movement across species lines [2]. In the last three decades, an estimated 75% of emerging human infectious diseases are zoonoses [3]. Additionally, contamination and pollution of the environment has affected its health and sustainability. Environmental degradation continues to create favorable settings for the expansion of existing infectious and non-infectious diseases for both human and animal health [4]. In addition, man and animals are threatened by non-infectious threats such as toxins and chemical contaminants, like the endocrine-disrupting chemicals in the environment [5]. Whereas humans and animals are under threat of emerging and re-emerging infectious diseases and environmental degradation the existing public health systems in many countries, especially poor nations, lack the capacity to manage these needs [6]. Current public health disease surveillance and response systems have not been tailored to facilitate early detection and timely response to disease threats at the human-animal-environment interface [4].

## One Health Approach

The need to address threats from infectious diseases at human-animal- environmental interface necessitates collaboration across the sectors. The “One Health” approach is envisioned as a vehicle to facilitate inter- and cross-sectoral collaboration. One Health has been defined as the collaborative efforts of multiple disciplines working locally, nationally, and globally, to attain optimal health for people, animals, and environment [7]. One Health seeks to combat existing and emerging infectious diseases by strengthening diagnosis, surveillance, response and recovery directed towards natural or intentional threats such as, chemical, toxicological, or radiological hazards. From the inception of the One Health initiative, the call was for building inter-disciplinary bridges to health in a globalised world [7].

There are challenges to the adoption of the One Health approach such as inadequate leadership and advocacy at national and sub-national levels and buy-in from stakeholders like clinicians, veterinary, industrial, and environmental professionals. Success in adopting the One Health approach will require overcoming many other barriers, including changing the mindset of healthcare providers from one of “disease care” to that of prevention, coupled with overcoming inter-professional prejudices amongst professionals from the different sectors.

A number of training programs designed to address disease threats from a preventive angle have been in existence for decades, however, none specifically addressed diseases that occur at human-animal interface. One such program is the field epidemiology and applied epidemiology training program (FETP). FETPs have a proven record of building a cadre of public health professionals that is able to address local public health challenges and the training curriculum can be adopted to address unique local challenges [8]. A case in point is the introduction of a laboratory track in African training programs referred to as Field Epidemiology and Laboratory Training Programs (FELTP) to build the national and sub-national capacity of public health laboratory services that are essential for multi-disease public health surveillance and response at national and regional levels. FETPs and FELTPs therefore provide a forum for advancing One Health in various countries where the programs are implemented.

## Integrating One Health into FETP/FELTP

FETPs and FELTPs are 2- year competency-based training programs based on CDC's Epidemic Intelligence Service model [9]. Their aim is to develop a public heath workforce capable of performing outbreak investigations, epidemiological research and surveillance [10]. The training is largely field based with trainees spending about 75% of their time working with health teams at district or provincial levels enabling trainees contribute to service delivery (e.g., identifying solutions to prevailing health problems) while the other 25% of the program time is dedicated to didactic sessions. The design of the training gives trainees an opportunity to understand the prevailing public health challenges at field sites and to interact with community members and professionals from other sectors such as veterinary services and water and environment. FETPs and FELTPs have been at the core of improving the health work force required to provide preventive and curative health care in sub Saharan Africa where the need for human resource for health is still felt [10,11]. The FETP/FELTP framework lines up well with the One Health approach which promotes collaboration and networking across the human, animal and environmental health sectors. Based on this shared ideology, FETPs/FELTPs provide an opportunity to advance the One Health approach through training which involves joint implementation of activities such as evaluation of surveillance systems, outbreak investigations by trainees from the different sectors. The outcome will be production of a workforce that is equipped to improve service delivery at various levels within the public health system and can advance implementation of multi-disease surveillance and response to disease outbreaks within the context of One Health. This will promote information sharing across the different sectors, thereby improving reporting and timely response to potential public health threats as required by WHO's International Health Regulations (2005) and Integrated Disease Surveillance and Response strategy (IDSR) [12,13].

Because the One Health practice is relatively new and has not yet been fully comprehended by many public health professionals at various national and sub-national levels both in curative and preventive health care, there is a justifiable need for integration of the One Health approach within FETPs/FELTPs since these programs have attained repute in effectively delivering applied/field epidemiology training resulting in a rapid expansion from four programs in 2005 to 12 FELTP programs spread across sub-Saharan Africa by August 2011. The goal of this strategy is to launch multi-sectoral collaboration with professionals from each of the sectors handling and responding to public health emergencies with a One Health mindset.

Field epidemiology training programs currently consist of the 2-year postgraduate FETP/FELTPs and the short term competency-based trainings such as the 2-week outbreak investigation short course targeting the frontline health workforce at different levels within the public health system. The trainings conducted in different regions benefit in-service workers from various sectors. The view is therefore to integrate One Health in the curriculum of the trainings.

### Practical demonstrations of One Health practice within FELTP

The Nigerian FELTP presents a good example of a FELTP designed on the One Health premise. The program aims to increase collaboration and strengthen linkages among epidemiologists and laboratorians from human and animal health sectors in the context of One Health. This collaborative effort is operational at state, federal and national levels. The multidisciplinary strategy is utilized to prevent, control and where possible eliminate infectious diseases within a larger ecological context of humans, animals, plants and environment [14].

Additionally, this program provides a unique stage where clinicians (medical doctors and laboratory scientists) train with veterinarians during didactics and in field assignments to respond to public health emergencies. By working in pairs, the trainees investigate and respond to disease outbreaks and environmental hazards arising from pollution and contaminants like lead. This model program has set the stage for collaboration, multi-disciplinary teamwork and information sharing across sectors created on a One Health foundation. This has improved public health multi-disease surveillance and timely reporting of potential health threats and notifiable diseases. In September 2010, trainees in the Nigerian FELTP were part of multi-disciplinary team that investigated lead poisoning in Zamfara state in North West Nigeria [15]. This was a unique public health problem that affected humans, domestic animals and the environment necessitating the application of the One Health approach in the control effort.

**Figure 1:**
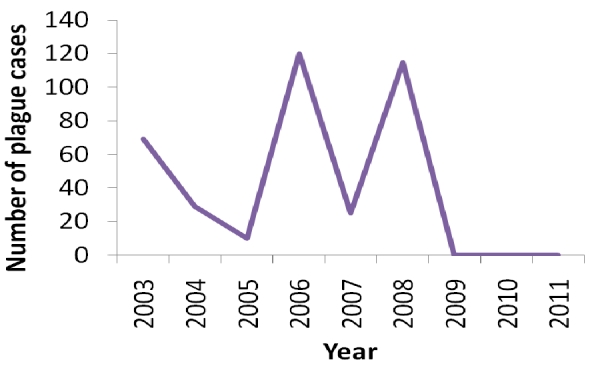
Trend of plague cases in Northwestern Uganda during outbreaks between 2003 and 2011

The One Health approach is yet to be integrated into the other African FELTPs. However, the programs have recognized this need due to looming threat of zoonoses like viral hemorrhagic fevers, brucellosis, and epizootics like foot and mouth disease (FMD) and are presently fast tracking the integration process. Some programs have participated in the investigation of zoonotic disease outbreaks and design of prevention programs. Graduates and trainees from the Uganda program were part of the integral multi-sectoral One Health team that responded to a plague outbreak in 2008 and participated in designing a comprehensive plague control framework that prevented plague outbreaks in north western Uganda in 2009 and 2010. This is a region that had been reporting and recording annual plague outbreaks in Uganda in the last decade [17]. As shown by the graph in Figure 1 below, there were no cases in 2009 and 2011.

The Uganda FETP trainees were also part of the multi-disciplinary team that responded to and investigated a yellow fever outbreak in northern Uganda between October 2010 and April 2011. The trainees were at the forefront of responding to an anthrax outbreak in wildlife in Queen Elizabeth National Park-Uganda between June and November 2010. They participated in rapid assessments for laboratory and surveillance capacity, training frontline health workers and community resource persons in anthrax detection and reporting existing, social mobilization and sensitization on anthrax prevention. The residents took lead in setting up One Health participatory disease surveillance monitoring systems for suspected anthrax cases in humans and livestock in communities neighboring the affected national park. The key achievement was the prevention of cross species spread of anthrax from the wild animals; there were no reported human or livestock anthrax cases.

AFENET together with CDC in 2011 started a 1 year post FELTP fellowship training, termed the AFENET CDC Emerging Pandemic Threats One Health Fellowship. The fellowship focuses on, skills building for FELTP alumni working in the government animal and human health sectors [16]. The fellowship seeks to advance the following One Health core competencies: outbreak investigations, surveillance, communication, research, leadership, computer skills, and teaching. Each fellow is required to take lead in conducting field assignments such as outbreak investigation, surveillance evaluation, analysis of surveillance data and writing manuscripts within the One Health context.

Fellows work in pairs (physician paired with either a veterinarian or an environmental health scientist) during the training. The training structure consists of 80% field work during which the fellows take a lead in setting up, evaluating, improving and monitoring disease surveillance systems in human and animal health sectors, investigating disease outbreaks in both sectors, analyzing surveillance data and One Health institutional building and improvement at sub-national levels (district, region/province) through training and supervision of front line health workers and surveillance officers.

The fellows are mandated to provide quarterly feedback of their field activities to their respective ministries and regional heads of public health and veterinary departments. The other 20% of the program is dedicated to didactics consisting of specialized short courses to broaden the fellows’ knowledge in science, advanced epidemiology, leadership and management as well as in participatory data collection and analysis techniques. Field sites comprise ministries of health and agriculture and related regional/provincial offices. Fellows work under the direct mentorship of highly experienced medical and veterinary epidemiologists from human and animal health sectors. The fellows work with their mentors to develop a plan of action addressing public health priorities of both human and animal health sectors with clearly defined timelines, monitoring indicators and deliverables. Fellows submit monthly reports to the fellowship office detailing their various field assignments. Additionally, fellows are required to submit abstracts to regional and international scientific meetings as well as manuscripts to a peer reviewed journal for publication before the end of the program. Other requirements for the fellowship include a thesis from each pair of trainees and a bound volume of their field work. The first cohort of fellows has graduates from Kenya and Uganda FETP/FELTPs. The fellows are government employees working in the human and animal health sectors who have been granted a one year study leave to participate in this full time program. It is anticipated that at the end of the 1 year training, the Fellowship graduates will become One Health leaders in their respective work stations and sectors at national and sub national levels. Recruitment of fellows is done with participation of the respective FELTP, government ministries of health and agriculture/livestock.

### Key achievements by the One Health Fellowship

The One Health Fellows designed and implemented a novel surveillance system for a mass gathering at the June 3rd Uganda Martyrs’ Day celebrations held annually in Uganda. In 2011, the Martyr's Day occurred after an Ebola outbreak was detected in Uganda. The novel system was able to detect suspected cases of viral hemorrhagic fever and these individuals were promptly isolated and investigated, luckily none of them had Ebola. The experience in developing and implementing this public health surveillance system informed Martyr's Day organizers and government of potential public health hazards that are likely sources of disease in the environment. This innovation was recognized by the Uganda Ministry of Health as the launch pad for applying surveillance during similar events that draw large masses of people.

This fellowship program has also taken lead on behalf of Uganda's Ministry of Health to investigate and respond to other public health emergencies. The fellows investigated the public health effects of lightning strikes which were widespread in Uganda between May and June 2011. The strikes were associated human and domestic animal deaths as well as destruction of property like homes. The findings of the survey informed the government on the magnitude of the fatalities, the need to strengthen lightning control and prevention measures like designing and disseminating public health messages about lightning.

The fellows are an integral part of the national multi-disciplinary rapid response team. This team has members from ministries of health and agriculture; wild life, disaster preparedness and other stakeholders like CDC, WHO, and UNICEF country offices and other international and local non-governmental organizations with a mandate to promote public health. The fellows have in this context worked with the national rapid response teams to investigate major disease outbreaks such as the 2011 Ebola Viral Hemorrhagic Fever in Uganda. The fellows are active members in the routine outbreaks monitoring meetings convened by the national multi-sectoral rapid response task force.

The fellows from Kenya are working on improving the surveillance system for zoonotic diseases among the animal handlers in abattoirs in Kakamega Province in Western Kenya. This followed findings from an evaluation of the existing surveillance system that indicated the need for improvement on the system so as to facilitate prevention, early detection and timely response to potential zoonotic disease outbreaks originating from the abattoirs.

## Conclusion

FETPs/FELTPs have played a significant role in strengthening public health multi-disease surveillance systems and response to disease outbreaks in Africa. The continent has been threatened by the emergence of infectious disease outbreaks having strong links with animals, both-domestic and wild while the occurrence of others like the vector-borne diseases has been driven by environmental degradation. The prevention and control of these emerging disease threats calls for modification of strategies geared towards multi- and cross-sectoral collaboration.

The One Health approach demands for collaboration across sectors at national and sub-national levels to strengthen surveillance and response to public health emergencies due to infectious and non-infectious causes such as chemical contaminants. The FETP/FELTP structures in different countries provide an excellent platform for integrating and adopting the One Health approach and practice. The positive impact demonstrated by FETPs/FELTPs which have adopted the One Health approach has proved feasible. This approach will contribute to early detection, timely reporting and response to all public health emergencies of international concern as required of member states by the International Health Regulations.
